# A Six-Tap 720 × 488-Pixel Short-Pulse Indirect Time-of-Flight Image Sensor for 100 m Outdoor Measurements

**DOI:** 10.3390/s26010026

**Published:** 2025-12-19

**Authors:** Koji Itaba, Kamel Mars, Keita Yasutomi, Keiichiro Kagawa, Shoji Kawahito

**Affiliations:** 1 Graduate School of Science and Technology, Shizuoka University, Hamamatsu 432-8011, Japan; 2 Research Institute of Electronics, Shizuoka University, Hamamatsu 432-8011, Japan; 3 Faculty of Science and Technology, Shizuoka Institute of Science and Technology, Fukuroi 437-0032, Japan

**Keywords:** iToF, range measurement, multi-tap, short pulse, subframe, range shift, ambient light

## Abstract

Long-range, high-resolution distance measurement with high ambient-light tolerance has been achieved using a 720 × 488-resolution short-pulse indirect time-of-flight (SP-iToF) image sensor featuring six-tap, one-drain pixels fabricated by a front-side illumination (FSI) process. The sensor performs 30-phase demodulation through six-tap pixels in each subframe, combined with five range-shifted subframe (SF) readouts. The six-tap demodulation pixel, designed with a lateral drift-field pinned photodiode, demonstrates over 90% demodulation contrast for a 20 ns light-pulse width. High-speed column-parallel 12-bit cyclic ADCs enable all six-tap subframe signals to be read within 4.38 ms. This high-speed subframe readout, together with efficient exposure-time allocation across the five subframes, enables a depth-image frame rate of 10 fps. The multi-phase demodulation in SP-iToF measurements, operating with an extremely small duty ratio of 0.2%, effectively suppresses ambient-light charge accumulation and the associated shot noise in the pixel. As a result, distance measurements up to 100 m under 100 klux illumination are achieved, with depth noise maintained below 1%.

## 1. Introduction

To facilitate the broader adoption of time-of-flight (ToF) image sensors in applications such as autonomous vehicles, drones, and automated guided vehicles (AGVs), it is essential that these sensors are capable of accurate distance measurement over long ranges, even under direct sunlight.

In recent years, direct time-of-flight (dToF) technology has made significant progress, and numerous LiDAR systems with excellent long-range performance have been reported [1,2,3,4,5,6,7,8,9,10,11]. There are also reports of distance measurements exceeding a kilometer using technologies such as fiber arrays [1] and dithered depth imaging [2]. While dToF is a promising approach for long-distance LiDAR applications, achieving high range-image resolution under strong ambient light remains challenging. This is primarily because implementing per-pixel photon-processing circuits and multiple SPAD (single-photon avalanche diode) elements capable of handling large numbers of ambient photons is difficult to scale down in size.

Continuous-wave indirect time-of-flight (CW-iToF) systems, which employ continuous wave modulation, offer the advantage of high pixel resolution [12,13,14,15,16,17,18,19,20,21]. However, their measurable range is typically limited to several meters, and operation with small pixel sizes under direct sunlight remains challenging. By binning every 16 pixels in a 1.2-megapixel iToF image sensor, measurements at 10 m under 80 klux ambient light have been demonstrated, though the resulting range-image resolution is only QVGA (320 × 240 pixels) [19]. An imager with 16 × 16 pixels and resistance to ambient light above 150 klux has also been reported [22], but due to its very low pixel count, it is not included in the comparison in this study.

Given the difficulty of simultaneously achieving long-distance measurement, high ambient-light tolerance, and high range-image resolution with dToF and CW-iToF technologies, another type of iToF sensor—short-pulse iToF (SP-iToF)—has attracted considerable attention for high-definition LiDAR applications [23,24,25,26]. By employing low duty-cycle pulsed light emission and sensor operation with multi-tap gating, SP-iToF sensors can accumulate signal charge from short pulses while draining unwanted ambient-light charge during the off-state of signal gating throughout most of the modulation cycle. This enables strong resistance to ambient-light interference. Furthermore, by shifting the measurement range using multi-tap pixels and capturing multiple subframes to extend the measurable distance, VGA-resolution four-tap SP-iToF sensors have successfully achieved long-range depth imaging up to 20 m under ambient light conditions of 100 klux [26]. These demonstrations, however, have been conducted using high-reflectivity white panels. Although multi-tap SP-iToF sensors show promise for long-range LiDAR applications, no prior studies have addressed their application-level performance for 100 m-class distance measurement under direct sunlight (100 klux) with acceptable frame rates and realistic target reflectivity.

This paper presents the first implementation results of an SP-iToF image sensor designed for 100 m-class LiDAR. The 720 × 488-pixel SP-iToF sensor features six-tap, one-drain pixels with a 12.6 μm square pitch and a high-speed column-parallel 12-bit cyclic ADC. It achieves five-subframe readout to implement 30-phase demodulation for 100 m range measurement, while maintaining a frame rate of 10 Hz. The impact of target reflectivity—specifically 80% versus 10%—on performance is also discussed.

## 2. Multi-Tap Pixel Architecture

### 2.1. Pixel Structure for Photodiode and Demodulator

**Figure 1:**
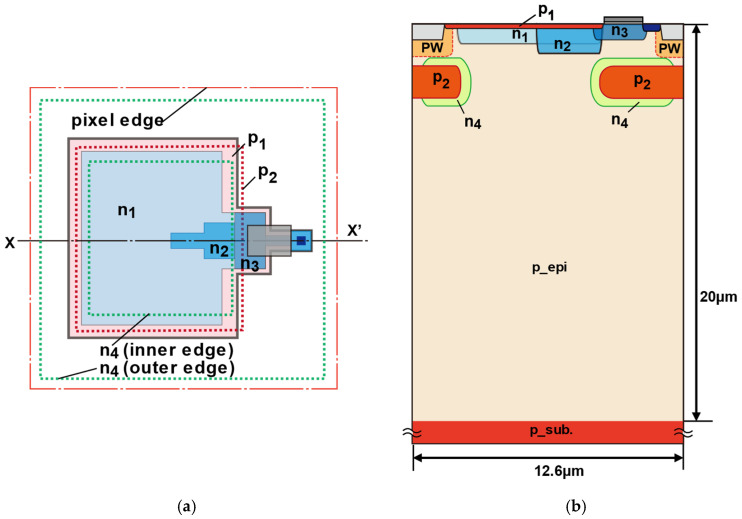
Basic pixel structure of the proposed sensor: (**a**) top view of single pixel; n_1_ to n_4_ represent n-type layers with different concentration profiles, while p_1_ to p_2_ indicate p-type layers, the three blue colors correspond to the regions of n_1_, n_2_, and n_3_, the green dashed line represents the edge of n_4_, the light red color represents the region of p_1_, and the red dashed line represents the edge of p_2_; (**b**) cross-sectional view (X-X′) describes depth distribution of each layer.

Figure 1 illustrates the fundamental structure of the photodiode and demodulator integrated into the designed SP-iToF sensor. The surface region of the photodiode adopts a lateral drift-field pinned photodiode architecture [24]. To establish a large and uniform lateral drift field across an extensive photodiode area, a triple n-type doping scheme—comprising n_1_, n_2_, and n_3_—is employed. Specifically:

(a) n_1_ is distributed across the front surface to cover the wide photo-sensitive region.
(b) n_2_ is positioned to generate a lateral drift field that guides photoelectrons from the central portion of the n_1_ region toward the vicinity of the modulation gate.
(c) n_3_ is located at the front edge and channel regions of the demodulation gate to enhance the potential modulation amplitude around the gate.

Near-infrared (NIR) light, commonly used as the active illumination source in ToF applications, exhibits a long absorption length in silicon. To efficiently collect photoelectrons generated in deeper regions and direct them toward the modulation gate, a deep diode structure composed of p_2_ and n_4_ layers is implemented. Additionally, a negative bias voltage is applied to the back substrate [27]. This deep diode configuration not only facilitates rapid electron transfer from the bulk but also serves to suppress hole current under negative substrate bias conditions. It also works as a charge shield to reduce the parasitic light sensitivity.

### 2.2. Six-Tap Demodulation Pixel

**Figure 2:**
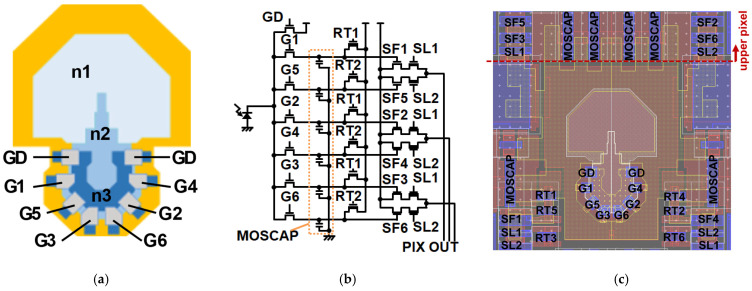
Tap placement and circuits within pixels: (**a**) in-pixel placement of charge transfer gates, drain gate, and n_1_–n_3_ region; here, the three blue areas correspond to the regions of n_1_, n_2_, and n_3_, and the yellow area represents the p_2_ region around the n-type areas; (**b**) in-pixel circuit, with each tap consisting of a gate, reset, source follower, and readout select transistor; (**c**) pixel transistor layout, where the upper transistors drive the pixel whose light-receiving region is located above this area.

In the proposed pixel design for long-range SP-iToF sensors, a pixel architecture featuring six-tap demodulation gates, as illustrated in Figure 2, is employed. To enable accurate long-distance measurements, the pixel must incorporate a large number of taps, exhibit high-speed carrier response to achieve strong demodulation contrast under short NIR light-pulse operation, and maintain high quantum efficiency with minimal parasitic light sensitivity [28]. As shown in Figure 2a, the six demodulation gates and associated draining gates are densely arranged within a compact area and connected to a large-area lateral drift-field photodiode. To suppress parasitic light sensitivity, the demodulation gates are placed at a distance from the photo-sensitive region. In contrast, the draining gates are positioned relatively closer to the photodiode to support efficient charge removal.

Figure 2b shows the equivalent pixel circuits. By controlling the voltage applied to the gate, signal electrons can be selectively directed either to a drain connected to the power supply for discharge, or to one of six taps connected to MOS capacitors for charge storage. This voltage-controlled modulation enables precise separation of signal paths, facilitating effective demodulation and accumulation of photo-generated carriers. The sensed six-tap signals are read out through six source followers and three parallel readout lines.

Figure 2c shows the location of transistors within the pixel. The capacitance of MOSCAP is 1.7 fF.

### 2.3. Pixel Potential Simulation

Since the absorption length of silicon is longer for near-infrared light compared to visible light, a 20 μm thick epitaxial layer substrate was employed to enhance quantum efficiency. Due to the lens-induced oblique incidence of light into the semiconductor, photo-generated electrons can be produced even at the deeper edge regions of the pixel. To ensure that these carriers are effectively collected as signal, the potential profile is designed to guide them toward the demodulation region. This design enhances carrier collection efficiency and contributes to improved demodulation contrast, particularly for electrons generated in regions distant from the pixel center.

**Figure 3:**
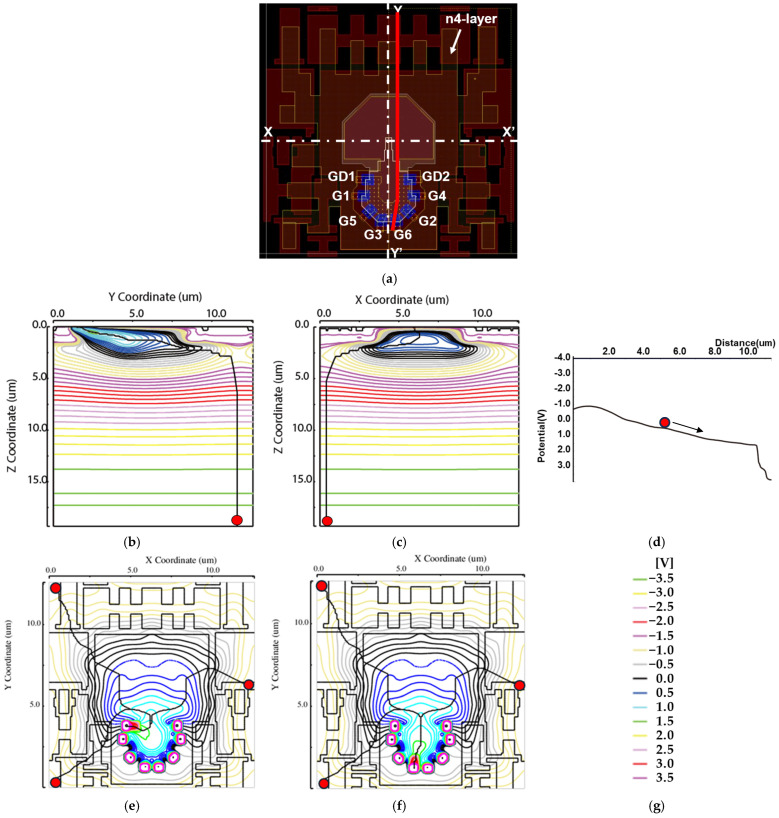
Potential simulation of pixel: (**a**) layout of single pixel; (**b**) charge transfer path to G3 in Y-Y’ cross-section when charge is generated at the red dot; (**c**) charge transfer path to G3 in X-X’ cross-section when charge is generated at the red dot; (**d**) potential slope of the Y–G3 path, along which the electron at the red dot flows in the direction of the arrow; (**e**) charge transfer path to GD1 when charge is generated at the red dot at GD1: high; (**f**) charge transfer path to G3 when charge is generated at the red dot at G3: high; (**g**) legend of potential contour lines.

Figure 3a shows the layout pattern of layers, including active regions (dark red), the three n-type doping layers described in Section 2.1 and Section 2.2, and deep n-type doping layer (n_4_), which have a special layout pattern to create a drift field to collect photoelectrons generated even at the pixel corners or within a few nanosecond to the surface central region of the photodiode. Further transfer to the vicinity of the demodulation gates is performed by other n-type layers (n_1_, n_2_ and n_3_).

Figure 3b,c show the simulated electric field lines in the X- and Y-sections, respectively. The paths of electrons generated in the deep region are drawn with black lines. Electrons transferred from the deep region to the surface by the substrate bias pass through the center of the pixel due to the effects of the n_4_ and p_2_ layers, and are guided to the gate region by the electric field generated in the surface n_1_–n_3_ layers. Figure 3d shows the potential formed by the n_1_–n_3_ layers along the Y–G3 path.

Figure 3e,f are simulated 2D potential plots on the xy-plane when the drain is set to high and all other gates are set to low, and the demodulation gate G_3_ is set high and all other gates are set to low, respectively. The solid black lines indicate the trajectories of electrons generated at three red-marked positions. Table 1 lists the transfer times for the three paths shown in Figure 3f. The worst transfer time of 2.1 ns is good enough for the SP-iToF operation using the light-pulse width of 20 ns.

## 3. ToF Measurement Method Using 6-Tap Pixels

### 3.1. Depth Measurement Algorithm in Single Subframe

**Figure 4:**
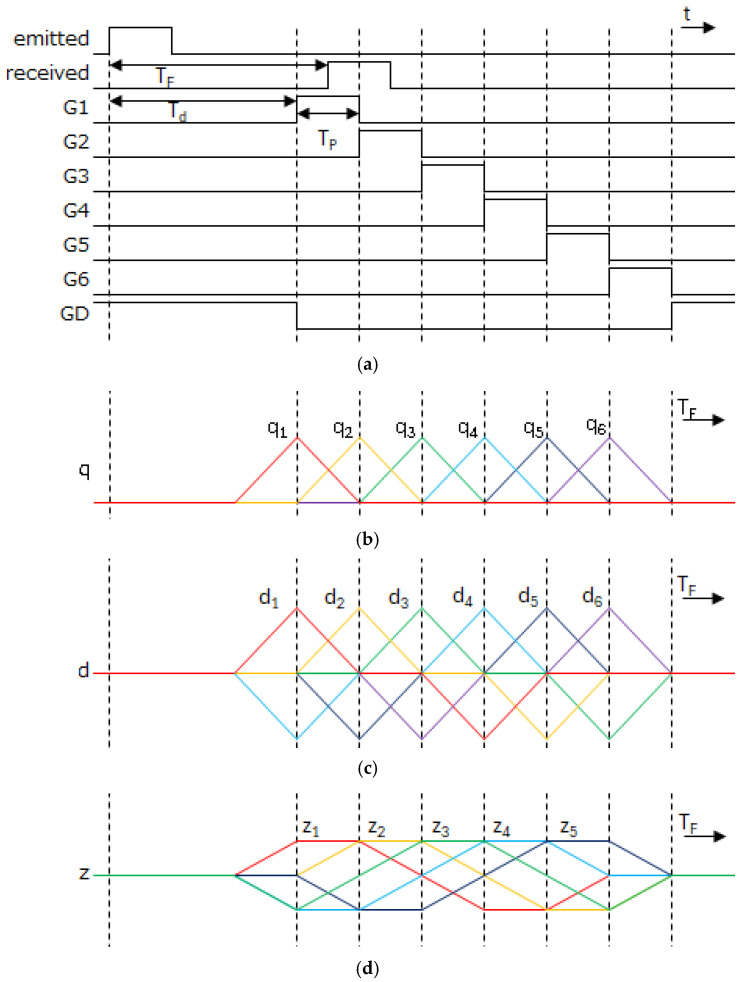
ToF measurement operation of 6-tap demodulation; (**a**) gated timing of G1–G6, GD, and light emission; (**b**) signal of each tap corresponding to reception timing T_d_; (**c**) differential signal between taps relative to light reception timing T_d_; (**d**) sum of signals between adjacent taps in gated timing.

Figure 4a,b illustrate the gate timing for each tap in single-subframe short-pulse iToF operation and the corresponding tap responses when reflected light is received at various timings, respectively. When the time of flight $T_{F}$ of the light varies from $T_{d}$ to $T_{d}+6T_{P}$, the signal electrons $q_{n}$ collected at the *n*-th tap exhibit a triangular waveform response, peaking when the time window of the tap aligns with $T_{F}$.

To measure $T_{F}$, in the range of 0 to $5T_{P}$, while suppressing background light interference by taking a difference between two taps, a function $d_{n}$ defined as

(1) $$d_{n}=\left\{\begin{array}{c} q_{n}-q_{n+3}\;(n=1,2,3)\\ q_{n}-q_{n-3}\;(n=4,5,6) \end{array}\right.$$

and a function $z_{n}$ defined as

(2) $$z_{n}=d_{n}+d_{n+1}\;(n=1,\ldots ,5)$$

are calculated. Then, the coarse $T_{F}$ defined as $kT_{p}$, where *k* is an integer, is measured by finding the index of $z_{n}$ that takes maximum value. Therefore, the determination of *k* is formulated as

(3) $$k=i\;(z_{i}=\max\left(z_{n}\right))$$

where *n* = 1,…, 5. The fine $T_{F}$ within each coarsely measured zone is calculated by $d_{n+1}/z_{n}$. Then the depth is expressed as

(4) $$Depth=\frac{1}{2}c\left\{T_{d}+T_{P}(k-1+\frac{d_{k+1}}{z_{k}})\right\}$$

where *c* denotes the velocity of light. The unit depth measured by one time window $D_{0}$ is defined as $(c/2)T_{P}$. In this single subframe case, the depth range of 0 to $5D_{0}$ can be measured.

### 3.2. Range Extention Using Subframes

**Figure 5:**
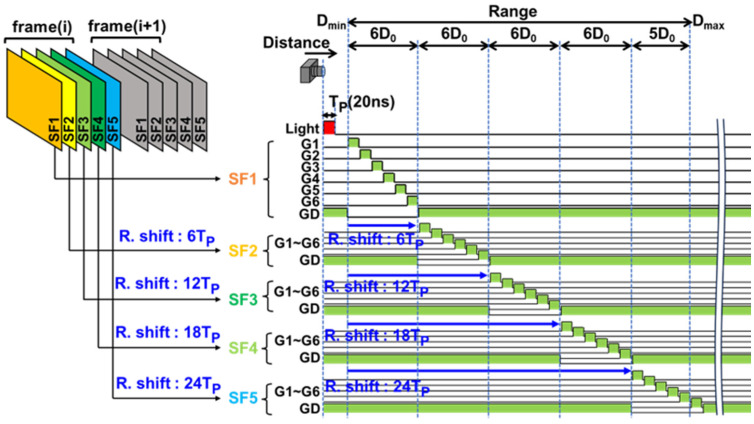
ToF measurements using range-shifted subframes.

A single-frame range image for long distance range is measured using multiple subframes captured together with the 6-tap pixel outputs in each subframe. Figure 5 shows the settings for subframe operations used for 100 m distance measurements. The gating time for the set of 6 gates in 5 subframes (SF1, SF2, SF3, SF4, and SF5) are shifted such that the delay time of the start of gating in SF1, SF2, SF3, SF4, and SF5 are $T_{d}$, $T_{d}+6T_{P}$, $T_{d}+12T_{P}$, $T_{d}+18T_{P}$, and $T_{d}+24T_{P}$, respectively. Note that though the ToF range of 0 to $5D_{0}$ is measured in the single-subframe case, the ToF range of 0 to $6D_{0}$ can be measured in each subframe in this multiple-subframe operation except for the subframe used for the furthest zone. Therefore, using a total of 30 time windows, the distance ranged from $D_{min}=\left(c/2\right)T_{d}$ to $D_{max}=D_{min}+29D_{0}$ and can be measured in this configuration. In the demonstration for maximal 100 m range measurement, $T_{P}$ is set to 20 ns, resulting in a measurable range by one time window. $D_{0}$ is approximately 3 m. By setting $D_{min}$ = 13 m, $D_{max}$ can be set to 100 m.

## 4. Measurement Results

### 4.1. Chip Specifications

**Figure 6:**
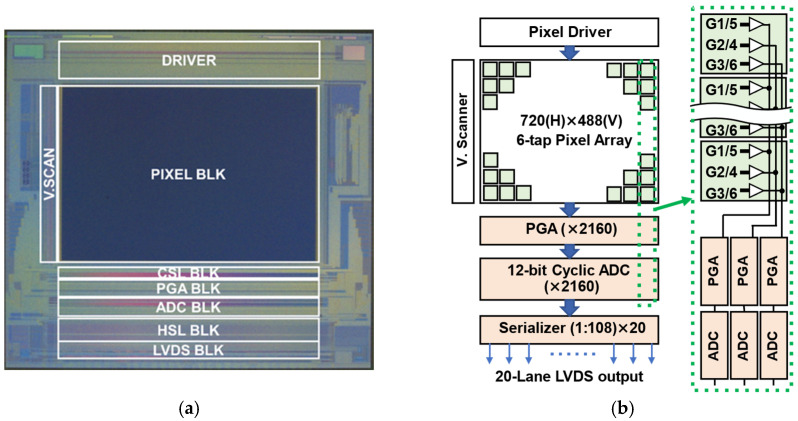
Prototype chip; (**a**) chip micrograph and diagrams of each functional block; (**b**) block diagram of the imager and readout circuit allocation for one column enclosed by the green dotted line.

Figure 6a,b illustrate a photograph of the implemented SP-iToF image sensor chip and its block diagram, respectively. The prototype chip was fabricated in a 0.11 μm CMOS process on a substrate with a 20 μm thick epitaxial layer. The 12.6 μm pitch six-tap pixels with a draining gate are arranged with a resolution of 720 × 488 pixels. The chip measures 9.1 mm × 6.1 mm.

The gate-driving pulses for the six-tap pixels with a draining gate are supplied by a 1D driver array located above the 2D pixel array. Each column with a 4.2 μm pitch in the column-parallel readout circuits includes a programmable gain amplifier (PGA) with selectable gains of 0.8, 2, and 4, and a 12-bit cyclic ADC. Consequently, to read signals from six taps, two cycles of three-tap parallel readout are performed, completing the readout for one pixel row. The cycle time, including reset and signal sampling of the pixel output, pre-amplification in the PGA, and 12-bit ADC conversion, is 4.48 μs. The outputs of the 2160 (=3 × 720) column readout channels are serialized in groups of 108 columns. The resulting 20-lane parallel outputs are further serialized into bit-serial signals, which are read out as 285 Mb/s LVDS signals. The overall data rate is 5.7 Gb/s, and the readout time for all subframe signals, consisting of six-tap signals from 720 × 488 pixels, is 4.38 ms.

Table 2 shows the specification of the implemented iToF imager. Since the pixel employs the floating diffusion (FD) node as the charge storage, the readout noise is dominated by the by kTC noise, resulting in a noise level of 41 electrons (e^−^). The measured quantum efficiency (QE) at 940 nm was 10.6%. This QE relatively lower than that expected for the use of a 20 μm epi layer is mainly because the microlens is not well optimized for the large-size pixel. It is expected that the use of advanced microlens technology and optimized and improved optical design for the pixel structure will enhance the QE to a maximum of 40% because the ideal internal QE of a fully depleted 20 μm thick epitaxial layer is approximately 40% at the wavelength of 940 nm.

### 4.2. Demodulation Characteristics

**Figure 7:**
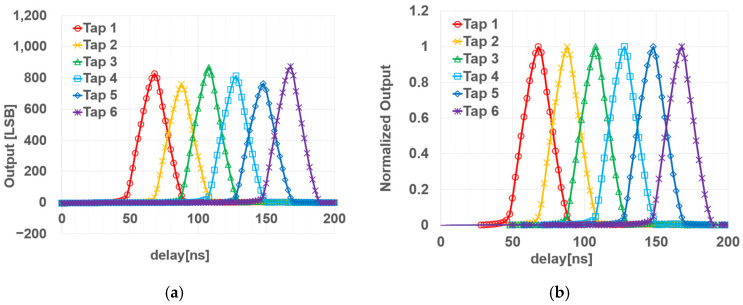
Demodulation characteristics of each tap; (**a**) tap signal corresponding to the light-source delay; (**b**) normalized tap signal to the maximum value of each tap.

To evaluate the demodulation characteristics of the six-tap pixel, a 940 nm square-topped light-pulse source with a width of 20 ns was used and captured by the sensor. Measurements were performed in 2 ns steps, covering a range from 50 ns before the time window of Tap 1 to 10 ns after the time window of Tap 6. Figure 7a plots the light-source delay on the horizontal axis against the output of each tap on the vertical axis, while Figure 7b presents the normalized output signals.

The demodulation contrast (*C_D_*) of the *i*-th tap is defined as the ratio of the peak signal intensity of the *i*-th tap, *S_i_*, to the sum of the signals from all taps at the delay time when the *i*-th tap’s intensity reaches its peak value, i.e.,

(5) $$C_{Di}=\frac{S_{i}}{\sum_{k=1}^{6}S_{k}}$$

Table 3 summarizes the demodulation contrast (*C_D_*) for each tap. The average *C_D_* across the six taps is 92.6%. Tap 1 and Tap 6 exhibit higher *C_D_* values, as there are no adjacent taps active in the preceding or following time windows, respectively. When excluding these two taps, the remaining taps achieve a high average *C_D_* exceeding 90%, demonstrating favorable demodulation performance.

### 4.3. Pixel Response Characteristics

To evaluate the response speed of the six-tap pixel, the sensor was illuminated with a short-pulse laser, and the pixel output was measured using a gating pulse width of 10 ns. An available laser light source with a pulse width of 69 ps (FWHM) and a wavelength of 851 nm was used for this evaluation. Since this light source (851 nm, absorption length in Si is 16 μm) generates a sufficiently large number of electrons in the deeper region up to 20 μm of the epi layer, it is useful for evaluating quantitative characterization of the pixel response as a time constant, though even better evaluation would be possible if a 940 nm ultra short-pulsed laser was available. The delay of the light source was scanned in 0.1 ns steps over a range from 5 ns before the time window of Tap 1 to 5 ns after the time window of Tap 6.

**Figure 8:**
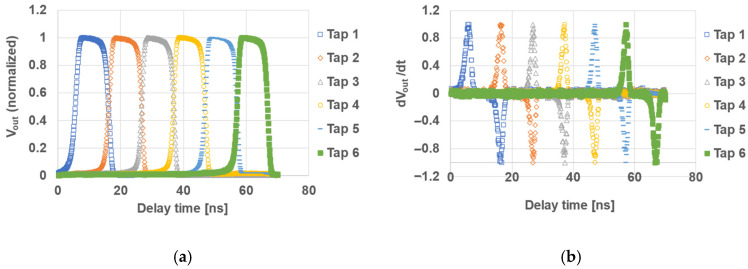
Response characteristics of pixels to incident 69 ps pulsed light; (**a**) tap output corresponding to the light delay; (**b**) the result of differentiating the tap output with respect to time.

Figure 8a plots the light-pulse delay on the horizontal axis against the pixel output (V_out_) on the vertical axis. Figure 8b shows the time derivative of each output (dV_out_/dt), illustrating the carrier response characteristics at the rising and falling edges of the gate windows. Table 4 lists the rise and fall response time constants for each tap as well as the FWHM. From these response characteristics, the measured FWHM indicates an average gate rise time of 1.74 ns and a fall time of 1.65 ns, with the slowest gate exhibiting a response time of 2.13 ns.

### 4.4. Outdoor Distance Measurement

**Figure 9:**
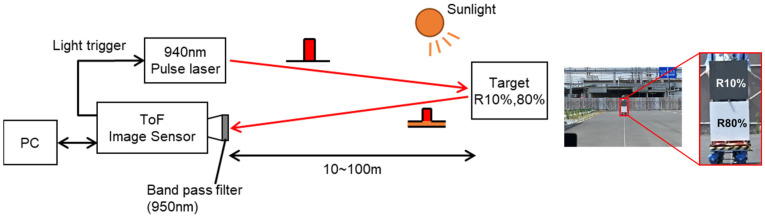
Outdoor range measurement setup. The red arrows indicate the emitted and reflected light.

Figure 9 illustrates the outdoor measurement setup under sunlight conditions. The camera equipped with the sensor is connected to a PC via an FPGA, which handles both camera control and data acquisition. A bandpass filter centered at 950 nm with a bandwidth of 50 nm is mounted on the camera. A near-infrared laser with a wavelength of 940 nm is triggered by the camera to emit pulses.

**Figure 10:**
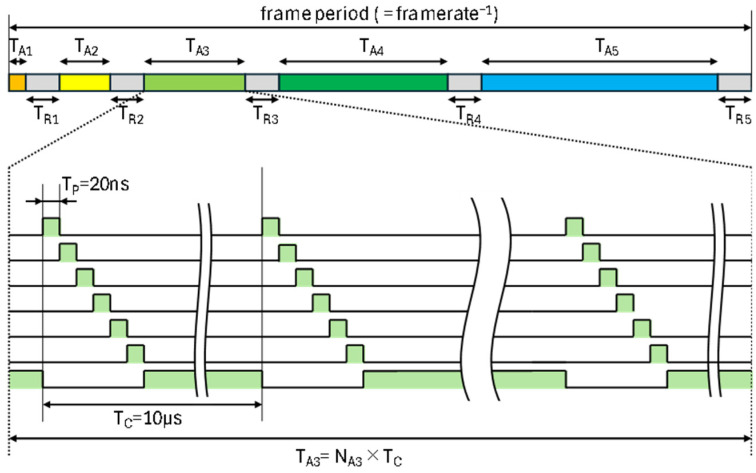
Timing diagram for demodulation operation. T_A1–A6_ represents the accumulation period of SF1-6, and T_R1–R6_ represents the readout period.

Operating conditions for six-tap pixel gating in each subframe and range-shifted subframe capturing using five subframes are illustrated in Figure 10. With the repetition cycle time for accumulation of 10µs, and the light-pulse width of 20 ns, the duty ratio of the light pulse is 0.2%. Based on the fact that the irradiated light attenuates inversely proportional to the square of the distance, the pulse gating numbers in the subframes are set such that the subframes used for covering closer distances have lower numbers and the subframes used for covering further distance have larger numbers [28]. The gating numbers used for the distance measurement and the resulting gating periods (signal accumulation time) are shown in Table 5. Including five subframe readout times, each of which is 4.38 ms, the entire frame period is 100 ms, achieving 10 frames per second (fps) for generating a depth image with a range from 13 m to 100 m.

The target consists of standard diffuse reflectors (500 mm × 500 mm) with reflectivities of 10% and 80% mounted on a cart and moved during measurement. Experiments were conducted both during daytime under sunlight exceeding 100 klux (up to a maximum of 110 klux) and at night under low-ambient-light conditions.

Table 6 lists the parameters of the light source and the camera lens.

**Figure 11:**
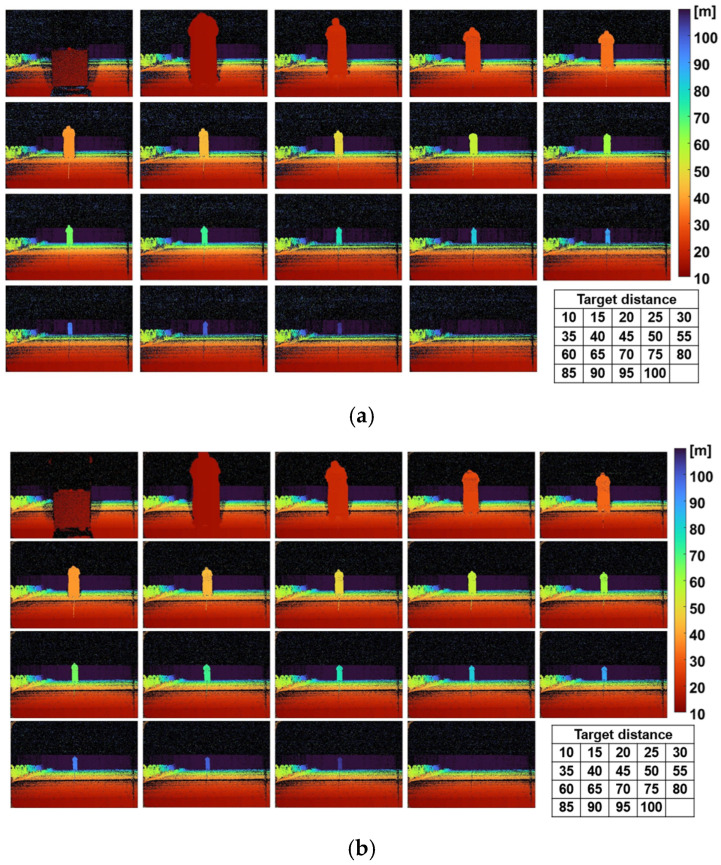
Range images when the target is moved in 5 m increments; (**a**) under sunlight over 100 klux; (**b**) at night.

Figure 11 shows the results of distance measurements conducted by moving the target in 5 m steps up to 100 m. Figure 11a presents the results obtained under sunlight conditions, while Figure 11b shows those captured at night. From these depth images with color mapping, it is evident that the panels at distances from 15 to 95 m are successfully measured. Since the panel at 10 m lies outside the measurable range of 13–100 m, its depth image should not appear. However, a false depth image of the panel with 80% reflectivity is observed. This issue is primarily attributed to pixel behavior when receiving an excessively strong light pulse, and will be addressed by re-examining the off-state potential barrier of the demodulation gates, which remains a subject for future work. The depth image of the panel at 100 m is not recognizable, as a wall located at approximately 101 m makes it indistinguishable from the wall’s depth image. Therefore, the target was successfully measured at all the distances under both sunlight and nighttime conditions.

**Figure 12:**
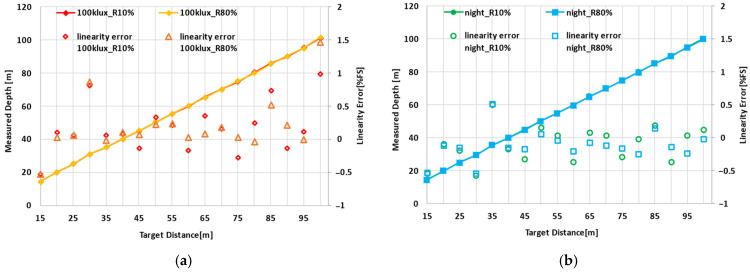
Measured depth and linearity error; (**a**) under sunlight over 100 klux; (**b**) without sunlight at night.

Figure 12 plots the target placement distance on the horizontal axis and both the measured distance and the non-linearity error between the measured and actual distances on the vertical axis. Figure 12a shows the results obtained under sunlight conditions, where the non-linearity error remained between −0.5% and +1.5% across the full-scale range for both 10% and 80% reflectivity targets. Figure 12b presents the nighttime measurement results, confirming that the error remained within ±0.6% for both 10% and 80% reflectivity targets.

**Figure 13:**
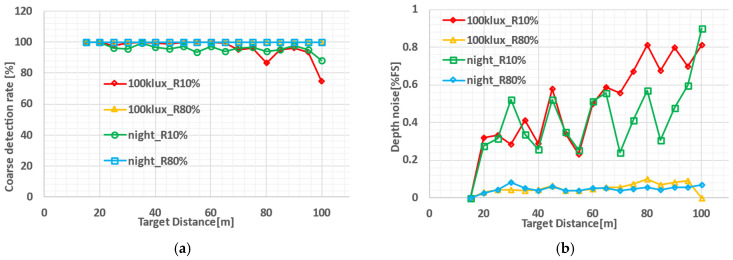
Detection rate and depth noise; (**a**) detection rate indicates the percentage of pixels capable of dToF-based ranging; (**b**) depth noise in pixels where dToF ranging is succeeded.

Figure 13a shows the coarse detection pixel rate (CDPR) at each target distance. Here, the CDPR is defined as the ratio of pixels that successfully measure the distance within an error range from −D_0_/2 to D_0_/2 to all the pixels to be measured. For the target with 80% reflectivity, a 100% detection rate was confirmed across all distances. Even under the most challenging condition—under sunlight at 100 m for the target with 10% reflectivity—the detection rate was 74.7%.

Figure 13b shows the depth precision or depth noise calculated from the pixels that successfully detected the object. For the 80% reflectivity target, a depth precision of less than 0.1% of the full-scale range (=100 m) was achieved. Even with the 10% reflectivity panel, the depth precision remained below 1.0%. The oscillation in the depth noise with distance is due to the pixel signal intensity modulation with distance in the proposed ToF measurement method using multiple subframes and multi-tap pixels.

**Figure 14:**
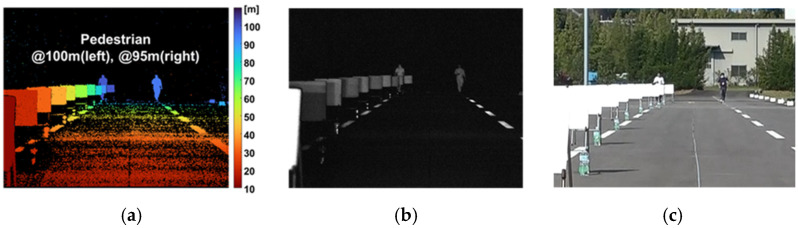
Captured image of pedestrian walking towards the camera under sunlight; (**a**) depth image; (**b**) the result of converting the tap signal into a grayscale image; (**c**) reference image taken with a color camera.

Figure 14 shows the results of capturing pedestrians positioned at distances of 95 m and 100 m. The imaging conditions were consistent with previous measurements, using five subframes (SF) and an exposure configuration that enables distance measurement up to 100 m at 10 fps. It was confirmed that pedestrians at both distances were successfully detected.

### 4.5. Comparison with Other Depth Sensors

Comparisons between the results of this study and previously reported ToF sensors operable under sunlight conditions (specifically those demonstrating usability in environments exceeding 70 klux) are presented in this subsection. CW-iToF and dToF sensors capable of operating under low-light conditions are available; they are excluded from the scope of this comparison [12,13,14,15,16,17,29,30,31,32]. In addition, a long-range binary-mode dToF imager with high ambient-light tolerance and 160,000 pixels has been reported [33]. Although high-resolution, however, it is also excluded from this comparison because its depth resolution is 7.5 m, which represents a unique specification.

Table 7 presents a comparison with other iToF sensors operable under sunlight conditions (specifically those demonstrating usability in environments exceeding 80 klux). When compared with CW-iToF sensors [18,19], the superiority of SP-iToF sensors in long distance measurement under strong-ambient-light conditions is obvious. This is because the acquisition of ambient-light charge in the SP-iToF sensor is greatly suppressed due to the very small duty of light pulse and unwanted ambient charge draining. The presented work demonstrated for the first time that the SP-iToF sensor realizes range imaging maximally to 100 m under strong ambient light, which is 5 times longer than the previously reported SP-iToF sensors [26]. Although a quantitative performance comparison between these is not applicable because the specification of the measurement conditions such as light-source power and target reflectivity are missing in the reference work, this advancement to reach to 100 m is mainly performed by using an advanced hybrid ToF measurement technique that uses the six-tap pixels with very low parasitic light sensitivity and high-speed column ADCs which allow to read five subframes to realize the 30-phase demodulations while attaining a frame rate of 10 fps.

Table 8 presents a comparison with dToF sensors operable under sunlight conditions (specifically those demonstrating usability in environments exceeding 80 klux). To compare these, we define a figure of merit (FoM) regarding the power efficiency of the light source. The relationship between signal photoelectrons, background photoelectrons, readout noise, and depth accuracy is expressed as [28]

(6) $$\sigma_{D}\propto \frac{D}{C_{D}}\frac{\sqrt{N_{a}+{N_{r}}^{2}}}{N_{s}}$$

where $\sigma_{D}$ is the depth noise, *D* is the target distance, *N_a_* is the number of photoelectrons due to ambient light, *N_r_* is the readout noise, and *N_s_* is the number of signal photoelectrons. Considering the effect of each parameter on the signal electrons, *N_s_* is expressed as

(7) $$N_{s}\propto R_{obj}\cdot P_{peak}\cdot R_{D}\cdot \frac{1}{R_{F}}\cdot \frac{{FoV}_{H}\cdot {FoV}_{V}}{N_{H}\cdot N_{V}}$$

where *R_obj_* is the reflectance of the target, *P_peak_* is the peak power of the light source, *R_D_* is the light-emission duty cycle, *R_F_* is the frame rate, and *N_H_* and *N_V_* are the numbers of the vertical and horizontal pixels, respectively. Then, we can define a figure of merit (FoM) regarding the efficient usage of average light power to achieve the targeted distance accuracy $\sigma_{D}/D_{max}$ under the given specification of target reflectivity and angular resolution determined by FoV and the number of pixels as

(8) $$FoM=\frac{\left[\sigma_{D}\right]\left[P_{peak}\right]\left[R_{D}\right]\left[FoV_{H}\right]\left[FoV_{V}\right][R_{obj}]}{\left[D_{max}\right]\left[R_{F}\right]\left[N_{H}\right]\left[N_{V}\right]}\times 100\;[\%\cdot n\;J{\cdot rad}^{2}]$$

When compared with dToF sensors operating at measurement ranges beyond 100 m, the primary advantage of this work lies in its exceptionally high angular resolution, which is approximately 115 times better than that reported in ref. [8]. However, the figure of merit (FoM) for efficient utilization of light-source power is about 86 times worse. In addition, the depth noise (or depth precision) for low-reflectivity targets is roughly nine times poorer than that of ref. [8], despite the difference in maximum measurement distance (100 m vs. 150 m).

Thus, the current status of this work can be recognized as a technology enabling long-range, high-resolution LiDAR imaging, albeit with less efficient light-power usage and only moderate distance precision when compared with dToF counterparts. It is also important to consider the difference in illumination methods: this work employs a flash-type approach, whereas dToF sensors rely on 1D scanning. Generally, light scanning provides higher efficiency in light-source power usage, while the flash type offers advantages such as simplified optics and avoidance of costly mechanical alignment between the FoV and the field of illumination (FoI).

In the following discussion, we address the future direction of this work, focusing on the adoption of scanning light sources and improvements in pixel-device performance to enable a fairer comparison with dToF sensors.

The first point concerns the scanning illumination method. Compared with the flash method, scanning reduces the exposure time per pixel to a fraction determined by the number of scan segments ($N_{\mathrm{div}}$), while increasing the light-power density per unit area by a factor of $N_{\mathrm{div}}$, assuming the overall average power remains constant. As a result, the contribution of ambient-light signals is reduced to $1/N_{\mathrm{div}}$, while the signal intensity itself remains unchanged. Consequently, scanning improves depth precision by a factor of $\sqrt{N_{\mathrm{div}}}$, or equivalently allows a reduction in light power by the same factor when ambient-light shot noise is dominant.

Recently, an SP-iToF sensor system employing a scanning VCSEL with 12 segments has been reported. Outdoor operation up to 27.5 m was demonstrated with an average power of 200 mW, clearly showing the enhanced light-power efficiency of scanning illumination even in SP-iToF systems [34].

The second point concerns pixel performance improvements achieved through advanced pixel structures and technologies. At present, the readout noise of 41e^−^ is dominated by kTC noise. This can be reduced to below 5e^−^ by employing kTC noise-cancelation techniques [35] or by introducing in-pixel charge storage to enable true correlated double sampling (CDS) [21]. Regarding quantum efficiency (QE), a three-fold improvement (to >32%) from the current pixel QE of 10.6% is feasible by adopting advanced process technologies such as backside illumination (BSI) and optimized microlenses. Indeed, an iTOF sensor using the latest BSI process has reported a QE of 38% [21].

Assuming the use of a 1D scan with 63 segments in the SP-iTOF system, together with improvements in read noise to below 5e^−^ and QE to 32%, the figure of merit (FoM) of the presented work can reach 0.088 [%·nJ·rad^2^]. This represents a 14-fold improvement, though it remains about six times worse than the dToF sensor reported in [8]. Nevertheless, if this 14-fold improvement is applied to reduce light-source power, the peak power can be lowered to 96 W—only about twice that of the dToF—while maintaining a precision of 0.9%. Since ToF sensor applications often require precision below 1%, this level is practically sufficient.

It can therefore be concluded that the proposed SP-iTOF approach offers a valuable solution for long-range, high range-image resolution LiDAR with a moderate yet practically adequate range precision of 1%. For example, in fully autonomous vehicles of level 4 or higher, detecting small but hazardous obstacles (e.g., a 10 cm cube) at 100 m requires an angular resolution of at least 0.03°. Using the dToF sensor of [8], which has five times worse angular resolution than this target, such obstacles are difficult to detect—though once detected, the distance can be measured with a precision of 0.1%. By contrast, the presented SP-iTOF sensor achieves twice the angular resolution required, ensuring reliable detection of obstacles, albeit with a precision of 1%. Clearly, the latter case provides a superior solution.

## 5. Conclusions

In this paper, we reported on the design of a short-pulse time-of-flight (ToF) image sensor for long-range outdoor imaging, along with an evaluation of its fundamental performance and experimental results under a 100 m measurement range and ambient illumination of 100 klux. The sensor incorporates six taps and one drain within each pixel. By employing short-pulse illumination and the six-tap architecture, the sensor captures six time-demodulated images per frame. Coarse time-of-flight measurements are obtained using a dToF-like method, which identifies the time-gated tap receiving the light pulse, while fine measurements are performed using an iToF-like method that calculates ToF from the signal intensity ratios between adjacent taps. With six taps and five subframes, the sensor executes a total of 30 demodulation operations, enabling depth imaging up to 100 m.

The prototype sensor demonstrated high demodulation contrast (>90%) with 20 ns pulse illumination. In outdoor depth measurement experiments under sunlight exceeding 100 klux, the linearity error ranged from −0.5% to 1.5%. The target detection rate, based on pixel count, was approximately 100% for targets with 80% reflectivity and 74.7% for targets with 10% reflectivity. The corresponding depth noise was 0.1% and 0.9% relative to the full-scale distance of 100 m, respectively.

Using a figure-of-merit (FoM) evaluation, current mainstream dToF sensors with 1D light scanning exhibit significantly better FoM than the proposed SP-iToF sensors. However, by adopting similar 1D light scanning and incorporating device performance improvements, the SP-iToF sensors can approach the performance of their dToF counterparts. In this context, the proposed SP-iToF sensors provide a promising solution for fully autonomous vehicles of level 4 or higher, particularly in detecting small but hazardous obstacles at 100 m, with a practically sufficient range precision of 1%.

## Figures and Tables

**Table 1:** Electron transfer time to tap 3.

Parameter	Path1	Path2	Path3
x coordinate at start	0.4 µm	0.4 µm	12.2 µm
y coordinate at start	0.4 µm	12.2 µm	6.3 µm
z coordinate at start	20.0 µm	20.0 µm	20.0 µm
Transfer time	1.85 ns	2.01 ns	1.50 ns

**Table 2:** Specifications of the iToF imager.

Parameter	Value
Process	0.11 μm FSI CMOS
Pixel Array	720 (H) × 488 (V)
Pixel Pitch	12.6 μm × 12.6 μm
Chip Size	9.1 mm × 6.1 mm
Epi. Thickness (=full well depth)	20 μm
Number of Taps	6 Tap, 1 Drain
ADC Resolution	12 bit
Read Noise	41 e^−^
Full Well Capacity	22 ke^−^
Dynamic Range	55 dB
Conversion Gain	34.2 μV/e^−^
Quantum Efficiency	23.8% @840 nm 10.6% @940 nm
Parasitic Light Sensitivity	0.003%
Power Consumption	0.6 W

**Table 3:** Demodulation contrast calculated by Equation (9).

Gate#	G1	G2	G3	G4	G5	G6	Average
C_D_ [%]	96.4	91.0	91.2	90.8	89.7	96.7	92.6

**Table 4:** Pixel response time constants for each tap and FWHM.

Gate#		G1	G2	G3	G4	G5	G6	Average
Time constant [ns]	Rise	1.02	0.88	0.74	0.83	0.78	0.72	0.83
	Fall	0.82	0.85	0.83	0.77	0.73	0.74	0.79
FWHM [ns]	Rise	2.13	1.93	1.50	1.69	1.67	1.81	1.74
	Fall	1.90	1.60	1.48	1.72	1.81	1.67	1.65

**Table 5:** Operating conditions for 100 m measurement.

Parameter	Value
Pulse width: T_P_	20 ns
Pulse cycle: T_C_	10 µs
Readout time: T_R1_~T_R5_	4.38 ms
T_A1_ (N_A1_)	2.23 ms (223)
T_A2_ (N_A2_)	6.61 ms (661)
T_A3_ (N_A3_)	13.3 ms (1330)
T_A4_ (N_A4_)	22.31 ms (2231)
T_A5_ (N_A5_)	33.64 ms (3364)
Framerate	10 fps

**Table 6:** Light source and optical lens used in the experiment.

Parameter	Value
Light source	940 nm VCSEL
Optical light power	2.1 W @average
Filter bandwidth	950 ± 25 nm
Lens focal length	50 mm
Lens F-number	1
FoV	10.4 degrees @Horizontal 7.1 degrees @Vertical

**Table 7:** Performance comparison of iToF depth sensors that mention use under sunlight (R: target reflectivity).

	This Work	[18]	[19]	[26]
Type	SP-iToF	CW-iToF	CW-iToF	SP-iToF
Process	110 nm FSI	90 nm BSI	90 nm/65 nm Stacked BSI	110 nmBSI
Pixel array	720 × 488	320 × 240	1280 × 960 320 × 240 (4 × 4 bin.)	640 × 480
Depth pixel pitch H × V [μm^2^] (area [μm^2^])	12.6 × 12.6 (158.67)	8 × 8 (64)	3.5 × 3.5 14 × 14 (4 × 4 bin.) (196)	5.6 × 5.6 (31.36)
Number of taps	6	Pseudo 4	2	4
Ambient light [klux]	100	130	80	100
Frame rate [fps]	10	10–60	n.a.	15
Maximum range [m] (outdoor operation)	100	4	10 (4 × 4 bin.)	20
Depth noise [r.m.s. % full scale]	0.9 (R: 10%) 0.1 (R: 80%)	0.54 (R: n.a.)	1.6 (R: n.a.)	1.3 (R: n.a.)

**Table 8:** Performance comparison of dToF depth sensors that mention use under sunlight (R: target reflectivity).

	This Work	[10]	[8]
Type	SP-iToF	dToF	dToF
Process	110 nm FSI	0.13 μm SPAD	90 nm/40 nm SPAD
Pixel array	720 × 488	1200 × 84	168 × 63
Lighting method	Flash	1D scan	1D scan
Depth pixel pitch H × V [μm^2^] (area [μm^2^])	12.6 × 12.6 (158.67)	12.5 × 89 (1112.5)	30 × 30 (900)
FoV [degrees]	10.4 × 7.1	24 × 12	25.2 × 9.45
Angular resolution [degrees]	0.014 × 0.014	0.02 × 0.13	0.15 × 0.15
Peak light power [W]	1350	n.a.	45
Ambient light [klux]	100	110	117
Frame rate [fps]	10	30	20
Maximum range [m] (outdoor operation)	100	200	150 (R: 10%)
Depth noise [r.m.s. % full scale]	0.9 (R: 10%) 0.1 (R: 80%)	0.15 (R: 10%)	0.1 (R: 10%)
FoM [% nJ rad^2^]	1.2	-	0.014

## Data Availability

Data are contained within the article.
